# Pandemic preparedness and responsiveness of research review committees: lessons from review of COVID-19 protocols at KEMRI Wellcome Trust Research Programme in Kenya

**DOI:** 10.12688/wellcomeopenres.17533.1

**Published:** 2022-03-03

**Authors:** Alex Hinga, Lisha Jeena, Esther Awuor, Jane Kahindi, Marianne Munene, Samson Kinyanjui, Sassy Molyneux, Vicki Marsh, Dorcas Kamuya

**Affiliations:** 1 Kenya Medical Research Institute (KEMRI) - Wellcome Trust Research Programme, Kilifi, P.O. Box 230-80108, Kenya; 2 Centre for Tropical Medicine, Nuffield Department of Medicine, Oxford University, Oxford, Old Road Campus, Headington, Oxford, OX3 7LF, UK; 3 Strathmore Business School, Strathmore University, Nairobi, Ole Sangare Road, P.O. Box 59857 – 00200, Kenya

**Keywords:** research review, ethics committee, COVID-19, Kenya

## Abstract

**Background**: The scale of the COVID-19 pandemic and novelty of SARS-CoV-2 presented unprecedented challenges in the review of COVID-19 protocols. We investigated how research at the Kenya Medical Research Institute - Wellcome Trust Research Programme (KWTRP) was reviewed, including by institutional and national level committees.

**Methods:** A document review and in-depth interviews with researchers, regulators and research reviewers were conducted. Documents reviewed included research logs of all protocols submitted between April-1-2020 and March-31-2021, feedback letters from review committees for 10 new COVID-19 protocols (n=42), and minutes from 35 COVID-19 research review meetings. Fifteen in-depth interviews were conducted with respondents purposively selected because of their experience of developing or reviewing COVID-19 protocols at the institution level (n=9 researchers, engagement officers and regulators) or their experience in reviewing proposals at a national-level (n=6 committee members). Data were managed and analyzed using MS Excel and NVivo12.

**Results**: Between April-1-2020 and March-31-2021, 30 COVID-19-related submissions by KWTRP researchers were approved. Changes to the review system included strengthening the online system for protocol submission and review, recruiting more reviewers, and trialing a joint review process. The turnaround time from submission to national approval/rejection over this period was faster than pre-pandemic, but slower than the national committee’s target. COVID-19-specific ethics questions centred on: virtual informed consent and data collection; COVID-19 prevention, screening and testing procedures; and the challenges of study design and community engagement during the pandemic.

**Conclusions**: The unprecedented challenges of the pandemic and added bureaucratic requirements created a more complex review process and delayed final approval of research protocols. The feasibility of conducting joint review of research during public health emergencies in Kenya needs further investigation. Consideration of the unique COVID-19 ethics issues raised in this paper might aid expedience in current and future reviews.

## Introduction

Since the onset of the COVID-19 pandemic in Wuhan China in late December 2019, there has been an unsurprising surge in publications and reports on the virus and its impact worldwide ^1, 2^. With over 250 million infections and over five million recorded deaths globally, the COVID-19 pandemic is arguably one of the worst health emergencies of this century ^3^. The conduct of research during health emergencies has been described by the World Health Organization (WHO) as an ethical imperative ^4^. Urgent research was and is required to inform pandemic responses, policies and vaccine roll-out strategies as well as best clinical management practices ^5– 7^.

Based on experiences from previous public health emergencies, it is clear that research review systems are central to expediency of key research questions needed to guide pandemic responses, but also that this expedience is challenged by the practical and ethical considerations that need to be made under pressure. Some of these challenges emanate from novelty of the pathogen, and thus the little data available to inform decisions about balance of risks, harms and burdens against benefits, and difficulties in determining appropriate standard-of-care, fair selection of participants, and the quality and legitimacy of informed consent ^8– 10^.

There is international ethics guidance available to inform research and research review in the context of the COVID-19 pandemic. One example is the 2020 Nuffield Council of Bioethics Report (NCOBR) on ethical issues in research during global health emergencies, which describes three core values to guide research ethics review; fairness, equal respect and reducing suffering ^11^. Another example is the WHO’s 2016 guidelines for management of ethical issues during infectious disease outbreaks, which recommends that research institutions prepare for health emergencies by instituting flexible regulatory and ethics review bodies, enabling pre-approval of protocols, and establishing joint review processes ^12^. Furthermore, the Council for International Organizations of Medical Sciences’ (CIOMS) guidance for the ethical conduct and review of research in developing countries and during health emergencies emphasizes the value of favorable risk-benefit ratio for participants, scientific validity, cultural sensitivity and rolling review of research protocols ^13^.

In addition, lessons learned from previous health emergencies and novel viruses could inform the conduct and ethics review of research during the COVID-19 pandemic. During the Ebola virus outbreak in Sierra Leonne, delays in ethics review and research approvals prevented the timely recruitment of participants into a Phase II treatment trial ^9^. The exclusion of pregnant women from vaccine trials during the Zika virus outbreak in South America and Ebola virus outbreak in West Africa meant that the impact of these vaccines on birth outcomes could not be determined ^14^. During the early stages of the HIV epidemic, placebo-controlled trials of antiretroviral treatment presented ethical tensions because the treatment was only available in developed countries ^15^. Also, and importantly, with increased pressure to conduct research in response to a health emergency there is risk of unintentional compromises on ethical standards ^9^.

Research review processes and experiences of researchers in Africa during the COVID-19 pandemic have received relatively little attention in the empirical ethics literature. Against this background, we investigated research review processes for COVID-19 studies developed and conducted by researchers at the KEMRI-Wellcome Trust Research Programme. This included investigation of protocol review turnaround times as a marker of expediency, ethical issues that were identified and how they were addressed, and research stakeholders’ perceptions and experiences of the preparedness and responsiveness of the review systems.

### The KEMRI Wellcome Trust Research Programme (KWTRP)

The KEMRI -Wellcome Trust Research Programme is based in the KEMRI-Centre for Geographic Medicine Research, Coast (KEMRI-CGMRC), which is one of the fourteen research centres of the Kenya Medical Research Institute (KEMRI). KEMRI, a state corporation established in 1979, has overall responsibility for providing leadership and guidance for health research in Kenya. The KWTRP conducts multidisciplinary research for health across four scientific departments – bioscience, epidemiology and demography, health systems and research ethics, and clinical research departments ^16, 17^. Research protocols at KWTRP are first reviewed internally by a Centre Scientific Committee (CSC) before submission to a national-level review committee ^18^ appointed by the National Commission of Science, Technology and Innovation (NACOSTI). NACOSTI was established via a parliamentary act (Science, Technology and Innovation Act No. 28 of 2013, a revision of previous Act 250), and has the responsibility to uphold quality in science, technology and innovation sector and to regulate the manufacture, trade and use of drugs in research respectively. It oversees all research conducted in the country and accredits Ethics Review Committees in Kenya.

### Review of protocols at KWTRP

**Figure 1.**
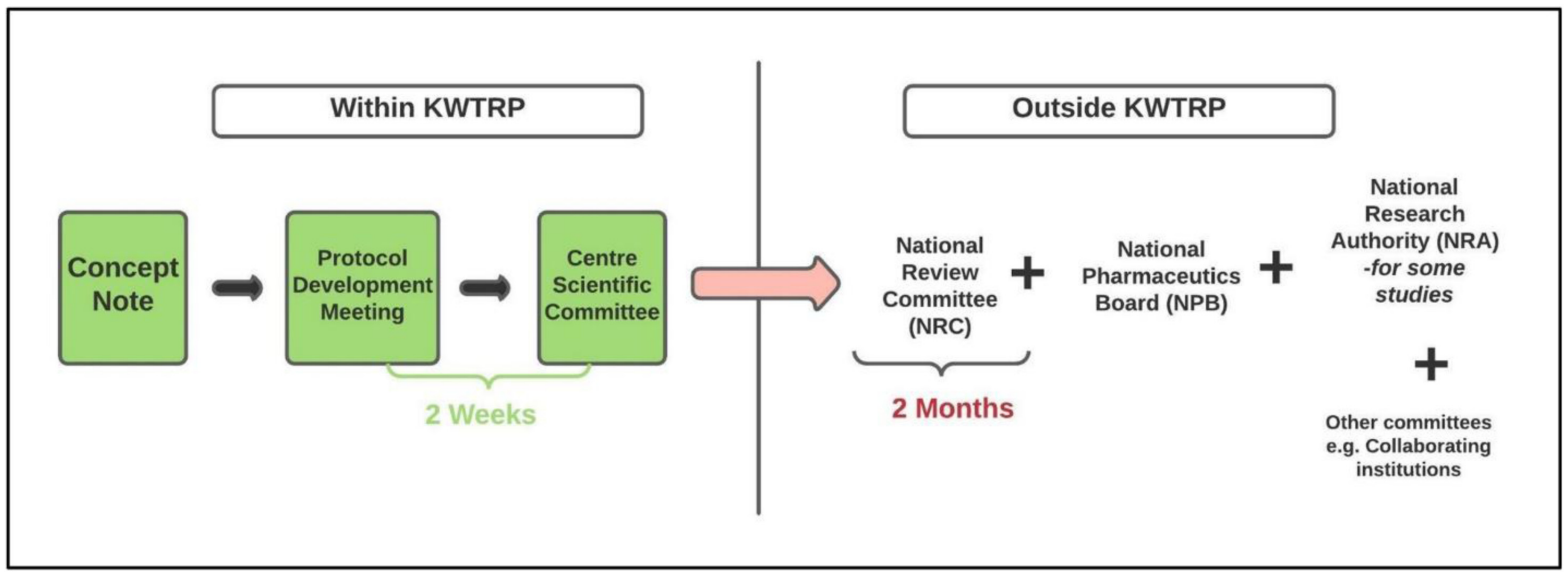
Review process for KWTRP protocols before COVID-19.

Under ‘normal’ (pre-COVID-19) circumstances, the review process at KWTRP involves three main steps. First, researchers circulate a concept note to CSC outlining the key aspects of their proposed research project, including project title and objectives. Next, a protocol development meeting is organized with the principal investigator where they receive initial comments and advice on their protocol from a team of CSC reviewers, including members of a sub-committee on consent and communication (CCC). Finally, the full protocol is discussed at the monthly CSC meeting where all reviewers and CSC members convene to decide on whether the protocol is locally appropriate, scientifically rigorous and ethically sound. It is then submitted to a national-level review committee (NRC), which reviews the scientific and ethical aspects of the protocol. Depending on the type of research, the protocol may also be reviewed by other regulatory bodies in Kenya such as the National Pharmaceutics Board (NPB) – in case of clinical trials – and National Research Authority (NRA). The review process for KWTRP protocols before COVID-19 is illustrated in Figure 1. The review and planning of research at KWTRP is often accompanied by translation of informed consent forms (ICF) as necessary while a community liaison group (CLG) is available to advise and support study teams in community engagement.

## Methods

### Study design

This empirical ethics study used a mixed methods approach; quantitative data drawn from research protocol logs and qualitative data collected through in-depth interviews and document reviews. Empirical ethics research draws on ethics frameworks and concepts and social science methods. It can include mapping out ethical issues, comparing ethics practice with ethical norms and recommending changes to ethical norms ^19^.

### Data collection methods

***Document and research logs review.*** The documents reviewed were selected to provide an overview of what research protocols were developed, received and reviewed over a one-year period since the first case of COVID-19 was identified in Kenya (i.e. April 1 ^st^ 2020 and March 31 ^st^ 2021), and the local and international guidelines that guided the reviews. The documents included research logs of protocols submitted to the KWTRP CSC between April 1 ^st^ 2020 and March 31 ^st^ 2021; feedback from KWTRP CSC and NRC on ten new COVID-19 protocols (n=42 review forms); minutes of sixteen KWTRP CSC and nineteen Research Coordinating Committee (RCC) meetings held between April-November 2020; and four guidelines developed by KWTRP and NRC before and during the COVID-19 pandemic as well as international guidelines described previously. Published documents were accessed online, while unpublished documents were provided by the KWTRP research office. Information extracted from the research logs included protocol title, name of principal investigator, departmental affiliations at KWTRP, and dates of protocol submission and feedback from the national-level ethics committee.

***Individual in-depth interviews.*** Individual in-depth interviews are a common method of data collection in qualitative research and are especially recommended where the aim is to co-create meaning and understanding of a complex issue ^20, 21^. Individual interviews were conducted by AH and DK between May and November 2021 to gain an in-depth insight into respondents’ perspectives on the planning and review of COVID-19 related research, and to explore issues identified through the document review. Purposive sample was used in selecting respondents ^22, 23^. All respondents had roles in undertaking COVID-19 research and/or review of COVID-19 protocols, and included principal investigators, members of the KWTRP CSC, national-level ethics committee reviewers, and members of the KWTRP Community and Policy Engagement team. The principal investigators (PIs) were purposively selected to include those that had the largest number of COVID-19 related protocols submitted for review. Many respondents had multiple responsibilities; for example, all KWTRP principal investigators were members of CSC with experience of reviewing KWTRP protocols. Some CSC members were also reviewers for the national ethics committee. The tool was piloted with researchers within the study team to check for clarity of questions.

The respondents were interviewed online using Microsoft Teams or Skype-for-business software. The interviews lasted 69 minutes on average (Min: 37 minutes, Max: 99 minutes, Median:71 minutes). All interviews were conducted in English and digitally recorded. All the digital recordings were deleted as soon as the data were transcribed and cleaned.

### Data management and analysis

KWTRP’s research log is in MS Excel format. The data for a one-year period was extracted and descriptive statistics generated, using MS Excel. This data included the number of COVID-19 studies per department, the type of submission (new protocol or amendment), and the number of days between protocol submission by principal investigators and final feedback. We also mapped out the specific milestones of the review process for one clinical trial. To ensure data safety, all files including documents and audio recordings were stored in a password-protected computer and only shared within the study team through the cloud-based and password-protected Microsoft OneDrive file storage and sharing service. Names and other identifiable details, such as specific roles and positions of individuals, were removed from documents and replaced with a number code to enhance confidentiality. Anonymity of respondents and other individuals involved in this study is maintained.

The CSC and NRC reviewers’ comments and interview transcripts were uploaded as MS Word documents into NVivo12 software (QDA Miner Lite is a freely available alternative software) and analysed using the framework analysis approach ^24, 25^. This involved iterative development of a coding framework, where two interview transcripts were selected and all the reviewers’ comments independently coded by authors LJ/EA/JK and LJ/AH respectively; and discussed with DK to harmonise any differences. The IDIs were separately coded and another thematic framework developed, with some of the themes similar to those developed from the review comments. In both frameworks, subcodes were introduced or removed as new data was added. Development of the framework was both inductive (informed by empirical data) and deductive (drawing on the ethics literature and guidelines). Data analysis identified key themes around the functioning, practical and ethical challenges for the ethics review systems during the COVID-19 pandemic and recommendations for strengthening research ethics review during pandemics.

### Ethical considerations

This study was reviewed by the KEMRI-Wellcome Trust Research Programme Centre Scientific Committee (CSC) and the KEMRI Scientific and Ethics Review Unit (KEMRI-SERU). It received research and ethics clearance from KEMRI-SERU (Ref: KEMRI/SERU/CGMR-C/054/4090) and the Kenya National Commission for Science, Technology and Innovation (NACOSTI) (License No: NACOSTI/P/21/9196). Additional approvals/permissions were provided by the Kilifi county government (KLF/DOH/RSRCH/VOL.1/83).

Potential interviewees were first approached by telephone or by email with an information sheet (Extended Data File 1) outlining the key features of the study including objectives, risks and potential benefits. In addition, an interview guide (Extended Data File 2 and 3) was emailed to potential respondents. All those who agreed to participate signed the consent form and emailed it to the interviewer. Before starting the digitally recorded interviews, the interviewer went over the consent information with the respondent and confirmed willingness to participate.

## Results

We begin by describing how the ethics review system at KWTRP functioned during the pandemic and the number and types of COVID-19 protocols reviewed. We then describe the changes made to the review system at KWTRP CSC and national-level review committee, the turn-around time for feedback and respondents' views and experiences. Finally, we describe the practical and ethical issues with new COVID-19 studies identified through the review system, respondents' views on how the review systems worked and recommendations on how they should be strengthened during public health emergencies.

### Summary of research protocols reviewed by KWTRP CSC and NRC during the study period

**Figure 2.**
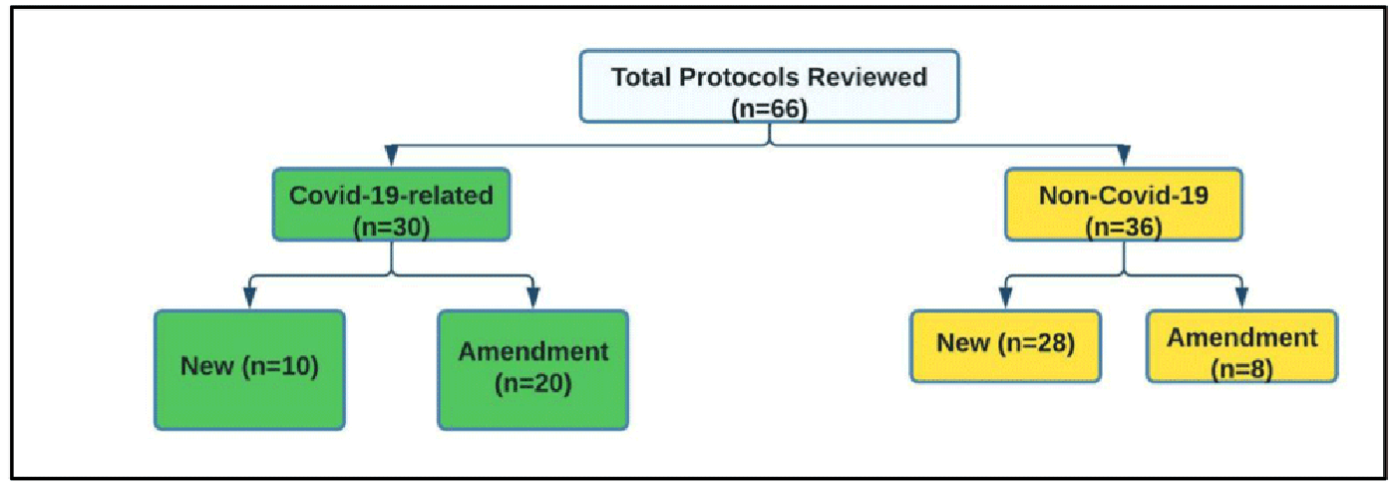
Number of COVID-19 and non-COVID-19 protocols reviewed for the period 01/04/2020 –31/03/2021.

Between April 2020 and April 2021, a total of 66 protocols were reviewed at KWTRP, of which 30 were COVID-19 related new protocols (n=10) and amendments (n=20) ( Figure 2). The 30 submissions were made by 22 principal investigators, with four researchers accounting for 41% of all protocols submitted.

**Figure 3.**
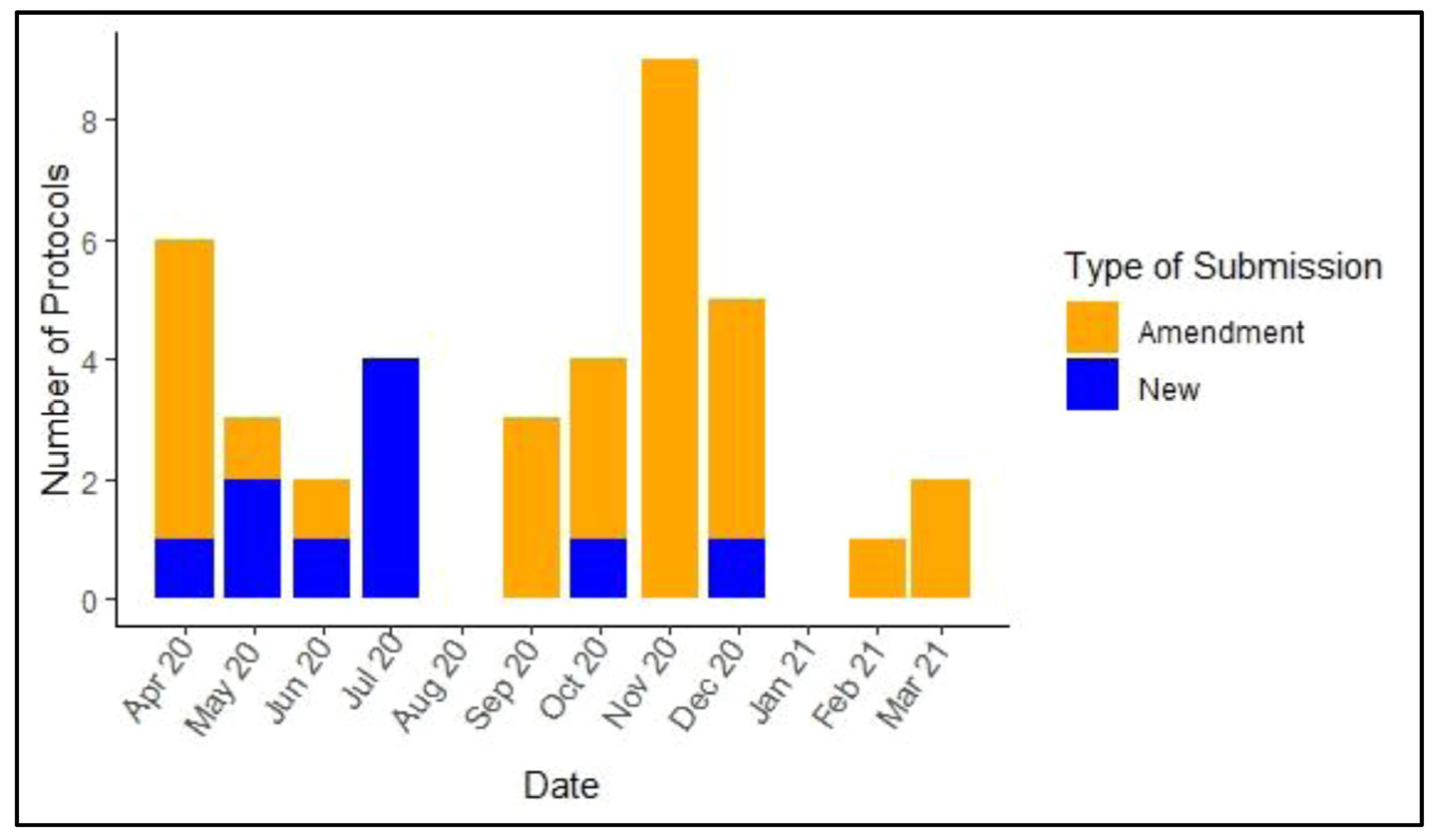
Distribution of new COVID-19-related protocols and amendments submitted in the period 01/04/2020–31/03/2021.

Eighty percent (8 out of 10) of new COVID-19 protocols were submitted for review early in the pandemic (April- July 2020). In the later part of the year (September 2020- March 2021), 61% (n=22) of submissions were COVID-19 related amendments to ongoing non-COVID-19 related protocols ( Figure 3). In the months of August 2020 and January 2021, no new submissions were made but there was ongoing review of previous submissions. Most (70%) of the new COVID-19 submissions were by principal investigators in the Health Systems and Research Ethics (HSRE) and Epidemiology and Demography (EDD) departments. However, research conducted at KWTRP is largely multi-disciplinary with co-investigators drawn from different departments.

### Structural and procedural changes to ethics review system during COVID-19

To respond to the uniqueness of the pandemic, several changes were introduced to the ethics review systems at KWTRP CSC and at the National Review Committee (NRC) to minimise personal interactions while also facilitating review of protocols. At KWTRP, a Research Coordination Committee (RCC) constituting senior scientific management staff was set up to provide oversight for the COVID-19 research agenda, coordinate response and support government. At the CSC committee level, a guidance document was developed by the CSC secretariat for reviewing COVID-19 related protocols. The NRC issued a COVID-19 guidance document, and accelerated its transition from a paper-based to an online *RHInnO* system ^26^. COVID-19 protocols were prioritised and the expected turnaround time for initial review by NRC was targeted at 14 days.

**Figure 4.**
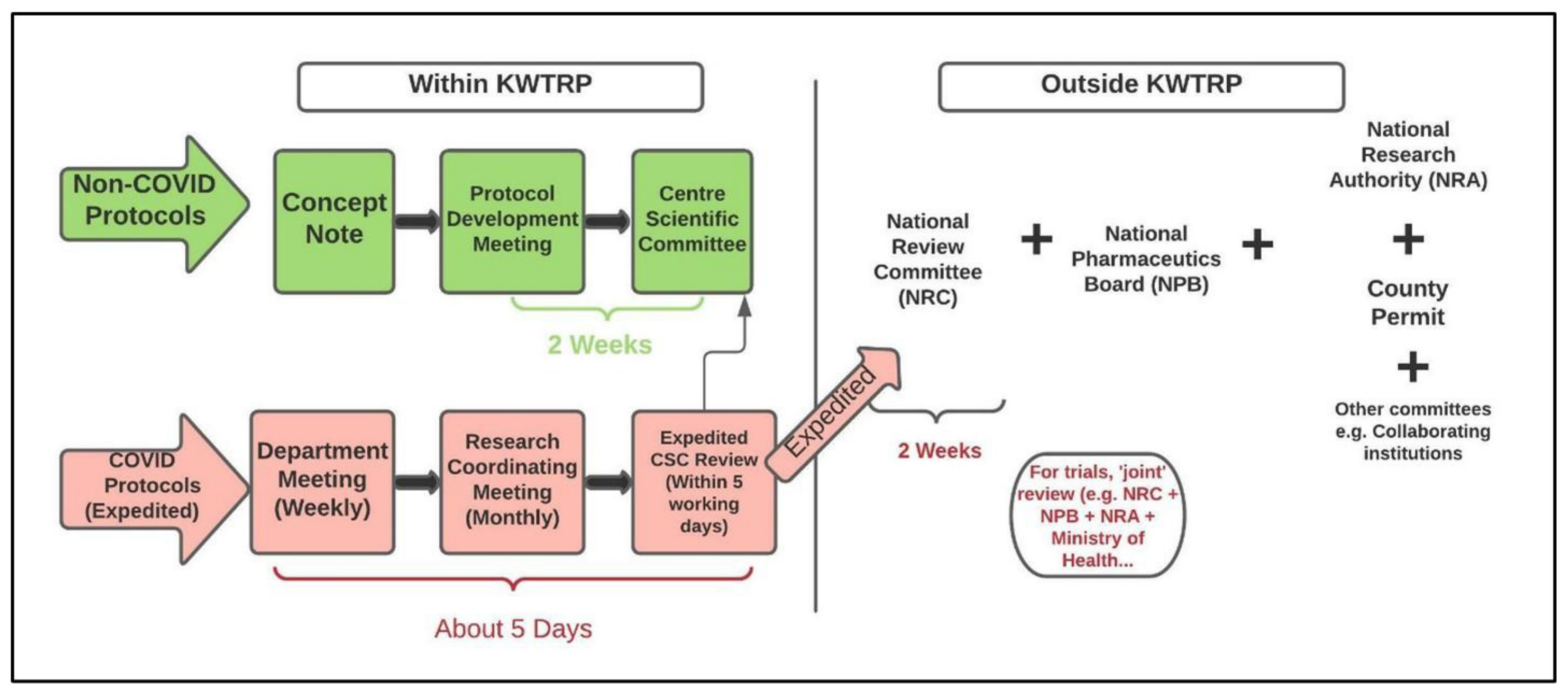
Adaptation of the review system internally at KWTRP and nationally during the COVID-19 pandemic.

New COVID-19 related legislature mandated a permit from NRA and from county government across all studies. Pre-pandemic, county permits were only required for community-based research projects. To expedite information flow and review of COVID-19 trials, a joint review was proposed – where the trial protocol would be discussed and reviewed by CSC, NRC, NPB and the Kenyan Ministry of Health at the same time. In practice, joint review was only attempted for one trial. Approval from NPB was specifically required for clinical trials. Review of non-COVID protocols resumed in August 2020 and followed the process outlined in Figure 1. Figure 4 summarises the procedural changes for review processes during COVID-19 pandemic with a focus on COVID-19 protocols and indicates the expected turn-around time for feedback from each of the review bodies.

**Figure 5.**
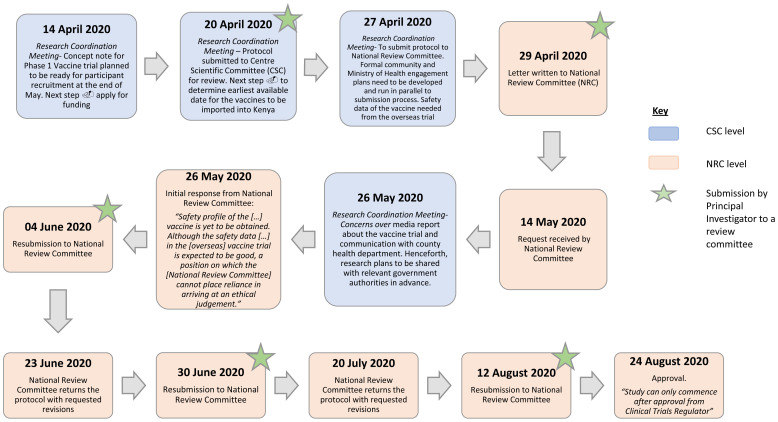
Flow Chart for turnaround time for clinical trial in Kenya from initial submission to approval.

To further understand and illustrate the changes that took place, we mapped the journey of a COVID-19 clinical trial through its review process. It took about four months from protocol submission to final approval by NRC, as shown in Figure 5 below. The figure shows several iterations of review and communication, indicating the complexity of review decision-making at the time, likely because of the paucity of data that was available about the novel coronavirus.

***Respondents’ views and experiences about the changes in the review systems.*** Most respondents supported the changes made to the review systems in response to COVID-19. They acknowledged that usual operations such as physical meetings had to stop to reduce risks of COVID-19 infections and to adhere to government directives on movement and social gatherings. They appreciated review committees’ efforts to establish an expedited review process, including through online meetings and submission of electronic protocols.

The shift from an office-based to a work-from-home environment was challenged by poor internet connectivity, family disruptions and a lack of resources at home (e.g. laptops, airtime, data bundles, spaces to work from). This was compounded by an increased workload on reviewers, probably due to the surge in research protocols submitted. To correct for these challenges, CSC members were supported with laptops and data bundles which made the transition smoother for them.

NRC reviewers reported facing many challenges, particularly in the early stages of the pandemic as the committee did not have the online infrastructure readily available; and had to make significant changes to adhere to the government directives of working from home.

“It was a very challenging time for us and when COVID happened initially, NRC was closed … and the operations were stalled. Everybody went home when the government announced that now ‘the country is on lock down, there is cessation of movement, employers should ask their employees to work from home’ … so NRC was closed.” IDI10

Although the NRC was able to transition to online systems during the pandemic, which was welcomed by all respondents, it was also generally considered to be overdue and to contribute to delayed timelines. There were also practical challenges experienced in moving to the online system, such as syncing the NRC online systems to KWTRP’s online submission system.

“There is also no way to link it [NRC online system] to our tracking processes. So there is no way to […] track and to see the progress of, you know... are you getting your reviews? Are there any screening comments?” IDI04

Both NRC and CSC developed guidelines for research review processes during the COVID-19 pandemic. Whilst the CSC guidance were developed fast and were available within a month of COVID19 reported in Kenya, the NRC guidance documents took around three months, reflecting the complexity in mandates of these committees (CSC institutional and NRC as national) and of their roles, particularly amid a pandemic.

“… So, we [NRC] developed a whole new set of guidelines for informed consent, for recruitment, for non-recruitment, for pausing and for [re-starting] we put all those guidelines there. So, for about four months many studies paused so that they could now look at our guidelines and see how they were going to proceed … the guidelines came out in June [2020].” IDI11
“For [CSC] the guidelines we were developing were centered on a process for expedited review and dealt mostly with submission and timeline, so it was more like a standard operating procedure. We were not looking at the ethical or scientific aspect of how to conduct studies in the context of a pandemic which largely is what these other guidelines were focusing on.” IDI04

Across the two committees, the guidance documents prioritized review of COVID-19 protocols. This was expected given that research was needed urgently to inform on the pandemic. In addition, as there was little information about the novel COVID-19, more reviewers were allocated per protocol than usual; at CSC reviewers per protocol were increased from 2 to 4 and the protocol development meeting was skipped. For clinical trials, pre-submission meetings involving the PI, CSC secretariat, NRC and NPB were held. These helped to discuss areas that were unclear and implications of some of the data gaps for the protocol towards expediting the review process.

“Initially we [NRC] only did COVID protocols, we introduced a quick turnaround track where [it would take] ten working days from the time that [we received a protocol] to when we communicated initial verdict from the committee. We had allocated ten reviewers per protocol but later we reduced back to three reviewers… and it is specifically for COVID protocols that were clinical trials that we gave so many members to review, but for the others, like if you had social science… if you are trying to find out the impact of COVID-19 maybe on travel or certain work… those ones we [allocated] three reviewers.” IDI14
“The protocol development meeting was dropped to save on time but still being aware that a lot of eyes had been looking at these protocols and giving guidance on how to develop them, so it [PDM] wasn’t a big step to lose. Also…, one thing we implemented for clinical trials was to have a pre submission meetings so that the protocol is looked at early enough by NRC and NPB and they can give comments on the issues that they feel need to be addressed or strengthened before the submission and we can also commit to some tentative timeline for review of the protocol. IDI04

### Turn-around time of review responses

From the document reviews, we calculated the turn-around time for all the new COVID-19 protocols that were submitted to the review systems between April 1 ^st^ 2020 and March 31 ^st^ 2021. The focus was on the time taken by NRC, as NRC is the final protocol approving authority for all research involving a KEMRI staff. Table 1 shows the time it took NRC to review a protocol and provide feedback, the time it took for researchers to respond to NRC feedback and the overall time to approval. The results showed that there was a 5-days delay (median of 19 days against expect turnaround of 14 days) in providing feedback for initial review of new COVID-19 protocols and a 10-day delay (median of 24 days) for amendment protocols respectively. The turnaround time reduced for subsequent iterations, with only a one-day delay in both. From the analysis, it showed that PIs took considerable time to respond to NRC’s comments with median of 24 days (range 0–87 days) for new COVID-19 protocols and 18 days (median of 0–51 days) for amendments. While there were delays in the review processes for COVID-19 research protocol, the review process was still faster than it was pre-COVID when it took two months to receive initial feedback and about four months for approval. 

**Table 1.** Turnaround time for new COVID-19 protocols and COVID-19-related amendments to ongoing research.

	New COVID-19 Protocols [Interquartile range]	COVID-19-related Amendments [Interquartile range]
	n= 10	n=28
1.Median number of days between first submission to NRC and first feedback from NRC	19 days [11 days; 56 days]	33 days [11 days; 56 days]
	n= 12	n= 13
2.Median number of days between subsequent revision submissions by PI and feedback from NRC (Excluding first submission)	17 days [6 days; 35 days]	16 days [0 days; 108 days]
3.Median number of days for PIs to respond to feedback from NRC	24 days [0 days; 87 days]	18 days [0 days; 59 days]
	n= 10	n= 28
4.Median overall time from first submission to NRC to protocol approval	83 days [43 days; 151 days]	45 days [7 days; 161 days]

***Respondents’ views on review timelines: expedited review and turn-around times.*** We investigated respondents’ views and experiences with the review timelines. All respondents seemed to appreciate the efforts made to provide feedback within a relatively short time, but also recognized that there were many challenges that might have contributed to delays. In interviews with review members, it was clarified that COVID-19 protocols were not in the expedited review category due to the novelty of the pathogen and the risks (and levels of the risks) that might be involved. Rather, they were considered as urgent requiring quick turn-around.

“So, COVID proposals were not in the expedited category. They were in the quick turn-around. During COVID … people are even sending studies about ICU, ventilation, breathing, oxygen, you know, things that were really very risky for the participants, but you see, we had to review them quickly.” IDI11

However, it seemed that principal investigators submitting protocols used these terms interchangeably to prioritize fast approval for COVID-19 related research. Subsequently, as expressed by one respondent who submitted a COVID-19 protocol to the national ethics committee for ‘expedited’ review, he/she did not experience any difference in feedback time to pre-pandemic submissions.

“NRC is an interesting one because they had the expedited review track. I paid for the expedited review, but I think it took the normal time it would take in the normal track [without expedited review]. So, I found that a bit interesting.” IDI08

This could have contributed to perceptions of delays in reviewing COVID-19 protocols as expressed by some PIs. Also, PIs were hoping for an even faster than pre-pandemic review process because they felt the research was urgent. Some respondents (CSC and NRC reviewers) attributed these delays to increased workload for reviewers, technical and organisational challenges and external interference rather than lack of ethics and research expertise to review protocols.

“…very quickly, I think, reviewers became overstretched. Obviously, even the working from home situation was not necessarily ideal for most people.” IDI05

Pronouncements by some French doctors about potentially exploitative research that could be undertaken during the pandemic raised the level of scrutiny for all research to ensure that it was indeed relevant to the population. Stakeholders who are not usually involved in review of protocols (e.g. MOH and County Governments) became more involved to safeguard the wellbeing and interests of the population, which could have contributed to further delays in protocol approvals.

“ What was happening in the country is that there was a public outcry that people are being used to test these drugs, so we had to try to balance the public interest… give the public confidence that whatever had been reviewed had been seen by as many committee members as possible.” IDI14
“We have the NRA approval …we have approvals from the Counties. There is a bit of an overkill. Because we are doing COVID testing we need approval from the Director General of the Ministry of Health, … so that one actually we haven’t received yet.” IDI08

A broader implication of delayed feedback, beyond the impact on the actual conduct and output of the research was the missed opportunity to showcase Kenya’s and Africa’s high-level capacity to conduct research to respond to a pandemic.

“…we were the first country in Africa to start engaging on this trial. But then South Africa came and overtook us… part of it might be attributed to that initial delay in approvals…Knowing that this vaccine has been evaluated by our researchers in [Kenya]…, there is something there that I think would have an indirect impact on... building public confidence in research… and you know to extend [that confidence] maybe even further to their response to national vaccine roll out programmes. We will never know because that did not happen.” IDI06

*Joint submission and review*

As we highlighted earlier, one protocol was submitted to multiple review and regulatory bodies at the same time. While the intention of the joint submission was to streamline and expedite the review and approval process, it might have added to the complexity of this process, as this researcher explained;

“After approval from the center scientific committee at KEMRI Wellcome, we made a joint submission to [NRC, NRA and a university review committee]. They were all viewing all the documents in real time, and so what then emerged of course is that everybody responds in their own different speeds which might reflect their capacity. Then you get into this situation where you are trying to respond to one committee and by doing that you find that you have already [revised the protocol] before the other committee responds.” IDI06

One participant felt that the protocol was subjected to a joint and lengthy review process because it was the first of its kind: it was the first clinical trial on COVID-19 in the country, public concerns were high about exploitative research during the pandemic and there was no precedent in reviewing such a trial in Kenya.

“I think the approval process for the first clinical trial was really delayed because I think there were other concerns beyond ethics… This led to administrative inertia of hesitancy in dealing with some clinical trial protocols. I remember that we had to have a joint committee meeting to consider the first … trial protocol… And that required an expanded meeting that had three committees, NPB, and all sorts of presentations just to consider one protocol many weeks after it had been submitted.” IDI05

While there was a joint submission and joint meetings to discuss the trial protocol, the different review and regulatory bodies did not make a joint decision on the protocol. One participant felt that there was a concurrent rather than joint review of the protocol.

“So for that one of different bodies, I think it has not happened in Kenya as far as am concerned. Some documents are usually sent to us and concurrently to NPB but we don’t review together as such. We have [attended] some online meetings where the investigators have explained the study and why they want us to have that concurrent [review]…what happens is that when NPB reviews they might recommend some changes to the project proposal that we had approved, so now it means the investigators also have to communicate to us again. I think joint review is something that can be explored.” IDI14

### COVID-19 protocols review: Issues raised and responses

Our analysis of review comments by CSC and NRC on the 10 new COVID-19 protocols showed that two-thirds of the comments were about ethical considerations while the remaining third were about regulatory processes and requests for additional information to enable a full assessment of the protocols. Table 2 summarises the issues that were raised by the CSC and NRC review committees, and the number of protocols that those issues were raised in.

**Table 2.** Summary of ethical considerations raised by CSC and NRC reviewers on new KWTRP COVID-19 protocols.

Type of issue	Number of protocols with issue raised	Summary of concerns raised by CSC and NRC reviewers’
** 1. Ethics considerations**		
Informed consent	10	- How was consent planned to be obtained in keeping with social distancing policies and implications of this on inclusion criteria? - Assessing comprehension of the consent process and the study virtually - Clearer information about the COVID-19 screening and testing procedures within the informed consent form - Requested that PIs use the KEMRI approved consent form template
Scientific Validity	8	- Sample size calculation and accounting for loss to follow-up in power calculation in a health emergency context - Generalisability of chosen study population - Protocols needing to be up to date with latest data available - Choice of diagnostic tools for the study design to be in line with COVID-19 in-country and international guidelines
Benefits and Risks to Participants	8	- COVID-19 specific risks such as the socioeconomic consequences and stigma associated with quarantine - Being more specific about potential side effects to medications and vaccines used in the trial - Compensation should align with KWTRP out-of-pocket expenditure guidance
Respect for Persons	8	- COVID-19 Risk mitigation strategies included in the trial - Confidentiality of COVID-19 test results, when can it be breached?
Fair Selections of Participants	8	- Provide alternative methods for inclusion of people without smart mobile phones or internet access - Translation of informed consent forms to the dialect of different ethnic groups - Inclusion criteria to be wary of generalised terms like ‘healthy adult volunteer’
Community Engagement	7	- More detailed community engagement plan beyond just feeding back information to communities
Data Sharing	4	- Informing participants about their data being used for future studies or at international laboratories
Collaborative Partnerships	4	- Building local laboratory capacity - Detailed list of sponsors and/or collaborators
** 2. Additional information requested by reviewers**		
Spelling, Grammar, Definitions	9	- Typographical errors - Study definitions and simplification of terms like *“suspected cases, confirmed cases and asymptomatic versus symptomatic individuals’’* and “ *respiratory tract virus”* and *“genetic finger printing”* were requested - Clarification of *“compensation versus reimbursement”*
Abstract/Lay Summary	8	- More detailed abstract/summary including all components of the protocol
Main protocol text: Methods	8	- Data collection, storage and waste disposal procedures (including training of staff) - Expected turnover time for test results - Clarification of target population for the study generally
Main protocol text: Hypothesis, Objectives	4	- Requested to improve clarity, and show how the methods meet the objectives
Main Protocol text: Limitations of the study	4	- Not enough detail about limitations, particularly limitations of conducting research in a pandemic
Main Protocol test: Budget	4	- Line item for COVID-19 prevention measures - Generally requested for more details and who will cover the costs e.g. quarantine/self-isolation
** 3. Regulatory processes**		
Missing Supporting Documents	4	- Ethics Certificate (either not attached or about to expire) - Study Insurance Certificate - Translation Certificates
Other review committees involved	2	- Requested a more detailed list of the names of sponsors, and other review committees, research institutions involved in multi-site studies

### Informed consent processes and community engagement

Issues related to informed consent (information in consent forms, consent seeking processes etc.) were raised in all the COVID19 protocols that were reviewed. Reviewers were concerned about the adequacy of informed consent processes in the absence of face-to-face interaction. Research protocols described varied informed consent processes including distributing hard copies of informed consent forms (ICFs) to respondents and seeking verbal consent over the telephone, requesting respondents to sign and email back electronic copies of ICFs and online/virtual consent. In response, reviewers sought explanations on how researchers would protect autonomy and ensure participants’ understanding of the proposed studies, as illustrated by this comment on a protocol by a reviewer;

“Would it be possible to have the respondents electronically fill the consent form rather than the study team do this? It seems to take away their autonomy when study staff do the check box ticking for them.” NRC Reviewer Comment

In seven protocols, reviewers requested researchers to clarify and give more details on how they would conduct community engagement in the context of a pandemic. The reviewers noted that community engagement plans described in the protocols - including strategies for adhering to COVID-19 mitigation measures while ensuring effective community engagement - were unclear. In response, researchers described ways in which existing community engagement mechanisms, including use of the existing channel of KEMRI-Community Representatives ^27^ would be engaged using virtual platforms, noting that this was not ideal but was a better option than no engagement at all.

### COVID-19 screening and testing

For the eight proposed studies that included COVID-19 screening and testing, reviewers emphasized the participants’ rights to detailed information about these procedures and the implications of test results. For example, reviewers noted that it would be important for participants to know whether a COVID-19 test would be mandatory and the procedures that would be followed if the test was positive. Also, reviewers wanted clarification on who would cover the costs of quarantine for participants who tested positive and how participants under quarantine would be compensated. Stigma from COVID-19 testing, regardless of a positive or negative result, was recognised as a risk of study participation. In one protocol, an NRC reviewer felt that it was insufficient for the researchers to state that *“[Stigma] is an ongoing risk for COVID-19 infection and not particular to the study.”* Given the government’s advocacy for testing and tracing potential COVID-19 cases, reviewers wondered how researchers would ensure confidentiality for study participants. Noting the challenges around screening and testing, reviewers suggested that proposed studies should offer pre- and post-test counselling and that disclosure of results to people other than the study participant should be discussed with the participant. Most researchers appeared to recognize these issues and agreed to take them on board, including working within an institutional/Programme-wide response system to the pandemic.

### COVID-19 study design

In eight protocols, reviewers raised questions on study design and methods, including sample size calculation, selection of participants and study populations and feasibility of data collection. For example, they noted that the pandemic was likely to disrupt healthcare seeking patterns affecting recruitment of study participants in healthcare facilities. In addition, they highlighted that the use of electronic and online platforms for seeking consent and data collection could unfairly exclude participants who did not have access to online and electronic resources. A related issue regarding study design was on how researchers would access and use new data about COVID-19 to inform study design and activities. As an illustration, one reviewer highlighted that a therapeutic agent for investigation in a proposed study had been suspended for use by WHO due to safety concerns. This statement was later retracted by WHO, allowing the study to continue.

### COVID-19 public health mitigation measures

In eight protocols, reviewers sought further information on how research staff and participants in the proposed studies would be protected from COVID-19 infections. Guidance on prevention of COVID-19 infection had been issued both within Kenya and by the WHO. However, reviewers highlighted that little information had been provided on the use of hand sanitizers, measures for social distancing and training of research staff on the use of personal protective equipment within protocols. This lack of detail could be due to changing regulations from the time of submission of a protocol, to the time of review. The recommendations made by reviewers were taken up by PIs and protective measures included in accordance with government regulations at the time.

### Respondents’ recommendations for strengthening review processes during pandemics

The recommendations here draw on respondents’ experiences of what worked well and what areas they felt needed improvement. While acknowledging the technical challenges involved, most respondents felt that the *online and electronic submission and reviewing of protocols* was a valuable change that should be sustained. They reported that online review meetings meant that reviewers could join at their most convenient venue and that meeting quorum improved. However, some noted that the depth of engagement and debate among reviewers decreased with online meetings and therefore suggested a hybrid system where there would be opportunities for online and in-person meetings.

A recommendation from across all the respondents was the need for *better communication and coordination* among ethics committees, reviewers and researchers. It was noted that there was particularly good communication and coordination within departments at KWTRP and between CSC and researchers; likely attributable to KWTRP being a well-resourced institution with strong pre-existing interpersonal relationships. Communication between CSC, NRC and researchers, however, was challenging. This was largely attributed to the IT challenges at NRC previously described. Researchers reported frustrations with different and sometimes conflicting review feedback from different review and regulatory bodies, given in a disjointed manner. This made protocol revisions quite challenging.

“But what happened here is that you have different bodies reviewing at different times and it’s that lack of being joined up in terms of that review process that just created some challenges in the response.” IDI06

Finally, respondents recommended that reviewers should be well supported and motivated and that guidelines and other guidance developed during the COVID-19 pandemic should be used for future responses. This could include some form of recognition and/or remuneration for reviewers’ hard work and use of pre-approved protocols and information material that could be adapted quickly to the specifics of a new public health emergency.

## Discussion and conclusion

Expedited review of research protocols during a public health emergency is crucial for a timely response to the emergency. This expedited review often requires changes to the review system to ensure that protocols are reviewed within a shorter timeline without compromising ethical standards. Some have shared their experiences of reviewing research protocols during the COVID-19 pandemic, including establishment of additional review processes for COVID-19 protocols, highlighting that such processes might improve the quality of research protocols and interdisciplinary collaborations but delay approvals ^28^. We discuss the key changes that occurred at KWTRP and national levels to expedite and strengthen the review process particularly for COVID-19 research, including the impact of these changes on turnaround times, and ethical issues identified through the review process. Some of these changes unintentionally increased the complexity of obtaining study approval. It is possible that the increased government involvement in regulating processes was in response to heightened fears of ‘exploitative’ research being conducted by international research bodies in Africa ^29^.

As outlined earlier, one KWTRP COVID-19 trial underwent a joint review by NRC. *Joint review is described as a way of streamlining different review processes* into a single comprehensive review with multiple bodies to expedite time to study implementation ^30, 31^. A process similar to joint review described for ethics review committees in the United States of America during COVID-19 was perceived to improve overall review efficiency ^32^. Other than the reported successes of the WHO’s Africa Vaccine Regulatory Forum ^33^, there is little evidence of joint review in previous health emergencies in low resource settings. The lack of joint review processes during the Ebola pandemic was seen as a missed opportunity to prevent the subsequent delays in review and approval ^34^. However, the joint review of the COVID-19 trial by NRC did not lead to a faster review and approval. Therefore, further research into the successes and challenges of the joint review process in low resource settings would provide insight into whether the process could be more widely implemented.

### Timing of review process and defining ‘delay’

During the Ebola disease outbreak of 2014 in Sierra Leone, the overall time in-country for review was at best 12 weeks ^30^. In comparison, the ethics review processes in Kenya during COVID-19 in the study period was faster, with 11 weeks overall to review new protocols and eight weeks overall for amendments. However, when comparing the turnaround time from initial submission to initial feedback, NRC took three times longer during the COVID-19 pandemic than the WHO Ethics Review Committee took during the Ebola outbreak ^34^. The COVID-19 treatment trial in the UK set a new precedent for turnaround time, requiring only nine days from study conception to first participant enrolment ^35^. These turnaround times during a health emergency can be used to gauge preparedness of researchers and review committees to respond to health emergencies timeously, while also recognising that it is only one aspect of this preparedness.

The KWTRP CSC and NRC compiled and published guidelines detailing COVID-19 related changes to review processes soon after the first case was reported in Kenya. Researchers developed protocols that covered a broad range of research questions with the intention of lessening the time taken for approval of subsequent amendments. As shown in the findings, amendments were approved quickly overall. This does suggest health emergency preparedness and responsiveness ^10^.

Capacity is another important contributor to enable expedited review. As per WHO recommendations, the CSC and NRC research ethics committees comprise of a diverse panel of reviewers from varying backgrounds and areas of expertise ^36^. However, in a health emergency where there is a rapid increase in workload for reviewers, expanding the base of reviewers working full time would be necessary to reduce reviewers feeling overstretched. Strengthening training and support systems for reviewers is a potential strategy for sustained improvements in research capacity ^37^. A unique unanticipated challenge of capacity building has been the need for strong IT capacity to support work-from-home. CSC and NPB were already at the forefront of this at the onset of the pandemic, and although slow at first, NRC did catch up, which is a positive marker of growth in local capacity in Kenya.

Unique to this research was that PIs also took a long time to respond to feedback from reviewers, which was possibly because they were also working out how to respond to some of the unique COVID-19 related questions posed by the reviewers – and/or were navigating similar administrative challenges and regulations faced by reviewers. The contribution of researchers to overall time to approval of protocols is not described elsewhere in the literature and could be explored in future research.

### Unique COVID-19 ethics concerns and its implications

Overall, the comments made by reviewers fell into similar categories as those described more widely in the research ethics literature ^13, 38^. However, how these issues emerged were often context specific, showing the importance of a grounded assessment of the nature of ethical issues in research, including identifying appropriate measures to counter these risks.

Some of the ethics issues identified in this study align with COVID-19 literature around virtual informed consent and the need to stay up to date with published data during an evolving pandemic to inform study designs ^32, 39^. During the Ebola outbreak, different ethics issues were raised in review of protocols such as on storage of blood samples for future use and the exclusion of pregnant women in clinical trials ^30^. This demonstrates that outbreaks can raise unique ethics concerns that are difficult to anticipate. A lack of precedent makes these novel areas challenging for reviewers and researchers to navigate and this could add to the delay in returning feedback to researchers and vice versa. Understanding these unique considerations and communicating them to researchers and reviewers as information becomes available could aid expedited review of protocols and amendments during the ongoing pandemic.

### Unintended consequences of the length of research review

Delayed review and approval of research protocols has broader unintended consequences. As observed with some of the COVID-19 protocols, delays may lessen local confidence in review bodies’ perceived ability to handle complex protocols; and potentially the confidence international research bodies have in the strength of review systems in Kenya. The WHO guidelines emphasises the importance of building public confidence and trust in the local capacity of research institutions to implement research quickly and efficiently ^12^. In the new era of rapidly shared information on social media platforms, delays in trial approval could be interpreted as an unsafe trial, which may affect trial participation or vaccine and therapeutic drug uptake once approved ^40, 41^.

Delays affecting non-COVID-19 research or the recommencement of trials that were stopped due to the pandemic has implications on the study participants enrolled in those studies, and on the research outputs. These unintended consequences of the changes, complexities and perceived delays in the research and ethics review system are important issues for consideration to inform current and future policies and practices within Kenya and beyond. 

## Data availability

### Underlying data

Data provided in the manuscript, including illustrative quotes, may be used without request but with reference to the full article and data. The authors confirm that, for ethical and security reasons, they are unable to make interview transcripts and internal administrative documents publicly available. As outlined in the consent forms, interview respondents were informed that the data would be shared without revealing individual identities and with other researchers after approval by relevant local and national review committees. Requests for these data can be sent to the coordinator of the KEMRI Wellcome Trust Data Governance Committee at (email: Data_Governance_Committee@kemri-wellcome.org). Access to these restricted data will be granted where deidentification can be adequately achieved to protect the privacy and confidentiality of the respondents and any mentioned individuals and institutions, and where the proposed use is seen as relevant to the nature of the data.

Harvard Dataverse: Pandemic preparedness and responsiveness of research review committees. https://doi.org/10.7910/DVN/WCJP86 ^42^

This project contains the following underlying data:

- Data File 1: KWTRP COVID-19 protocols submitted for review including turnaround times for review (.xlsx)

Data are available under the terms of the Creative Commons Zero "No rights reserved" data waiver (CC0 1.0 Public domain dedication).

### Extended data

Harvard Dataverse: Pandemic preparedness and responsiveness of research review committees. https://doi.org/10.7910/DVN/WCJP86 ^42^

This project contains the following extended data:

- Extended Data File 1: Informed consent form (PDF)
- Extended Data File 2: Interview guide for researchers (PDF)
- Extended Data File 3: Interview guide for reviewers (PDF)
- Extended Data File 4: Coding framework for documents (PDF)
- Extended Data File 5: Coding framework for interviews (PDF)
- Extended Data File 6: COREQ Reporting Guidelines (PDF)

Data are available under the terms of the Creative Commons Zero "No rights reserved" data waiver (CC0 1.0 Public domain dedication).
