# Supplementary material for: Pandemic preparedness and responsiveness of research review committees: lessons from review of COVID-19 protocols at KEMRI Wellcome Trust Research Programme in Kenya
Source: Wellcome Open Res. 2022 Jun 21;7:75. Originally published 2022 Mar 3. [Version 2] doi: 10.12688/wellcomeopenres.17533.2 (PMC9257264; doi:10.12688/wellcomeopenres.17533.2)
Supplement: Peer review history

# Peer review: Reviewer response for version 2

**DOI:** 10.21956/wellcomeopenres.19933.r51171

**Published:** 2022-07-05

**Authors:** Deborah Nyirenda

**Affiliations:** 1 Community Engagement and Bioethics, Malawi-Liverpool-Wellcome Trust Clinical Research Programme, University of Malawi, Blantyre, Malawi

No further comments to make. However, on page 9 'Turn around time of review processes', ...median of 24 days (range 0-87 days) ...and 18 days **(median of 0-51 days)**. Can the authors crosscheck if they meant to present a range of 0-51 days rather than median of 0-51 days?

Is the work clearly and accurately presented and does it cite the current literature?

Yes

If applicable, is the statistical analysis and its interpretation appropriate?

Yes

Are all the source data underlying the results available to ensure full reproducibility?

Yes

Is the study design appropriate and is the work technically sound?

Yes

Are the conclusions drawn adequately supported by the results?

Yes

Are sufficient details of methods and analysis provided to allow replication by others?

Yes

Reviewer Expertise:

Social Science, bioethics

I confirm that I have read this submission and believe that I have an appropriate level of expertise to confirm that it is of an acceptable scientific standard.

# Peer review: Reviewer response for version 1

**DOI:** 10.21956/wellcomeopenres.19387.r49043

**Published:** 2022-03-31

**Authors:** Deborah Nyirenda

**Affiliations:** 1 Community Engagement and Bioethics, Malawi-Liverpool-Wellcome Trust Clinical Research Programme, University of Malawi, Blantyre, Malawi

This article is reporting findings from a mixed methods study involving document reviews and qualitative interviews on ethical review processes of research protocols during the COVID-19 pandemic. I advise the authors to a) include a statement on positionality in the methods b) proofread the paper and clarify or correct the following:

 Page 3. During the early stages of the HIV epidemic, placebo-controlled trials of antiretroviral treatment presented ethical tensions because the treatment was only available in developed countries.- please clarify whether the ethical tensions arose because of the nature of placebo controlled trials or because treatment was only available in developed countries?

 Page 5.The CSC and NRC reviewers’ comments and interview transcripts were uploaded as MS Word documents into NVivo12 software (QDA Miner Lite is a freely available alternative software). It is not clear why they have talked about QDA Miner Lite being freely available when they used NVivo - please explain.

 The summary of research protocols reviewed by KWTRP is confusing & needs to be presented clearly. The abstract talks about 10 new COVID studies & 42 feedback letters. Table 1, indicates 10 new COVID studies and 28 COVID related amendments. Page 5, talks about 30 COVID related protocols (n=10). Please be consistent. In addition, they have indicated that 66 protocols were reviewed but the sample sizes for COVID studies or amendments don't add up to 66 - I suggest that they either provide COVID only studies or indicate the number of non COVID studies.

 Page 5 & 6, rather than talking about the departments that submitted protocols, readers may be more interested in an overview of the COVID-19 studies reviewed to appreciate the ethical issues presented thereafter.

 Page 6 & 9, the subtitles/themes are connected to the first paragraph/sentence.

 Figure 5, has symbols in the first 2 text boxes, immediately after Next step-include the meanings of these symbols in the key. 

 Page 9, it showed that PIs took considerable time to respond to NRC’s comments with median of 24 days (range 0–87 days) for new COVID-19 protocols and 18 days (median of 0–51 days) for amendments. Please check and ensure that the median in calculated properly. 

 Page 12, some words are in italics please check if this was meant to be. 

 Please ensure that COVID-19 and KEMRI-Wellcome Trust Research Programme are spelt consistently.

Is the work clearly and accurately presented and does it cite the current literature?

Yes

If applicable, is the statistical analysis and its interpretation appropriate?

Yes

Are all the source data underlying the results available to ensure full reproducibility?

Yes

Is the study design appropriate and is the work technically sound?

Yes

Are the conclusions drawn adequately supported by the results?

Yes

Are sufficient details of methods and analysis provided to allow replication by others?

Yes

Reviewer Expertise:

Social Science, bioethics

I confirm that I have read this submission and believe that I have an appropriate level of expertise to confirm that it is of an acceptable scientific standard, however I have significant reservations, as outlined above.

# Response

**Published:** 2022-06-14

**Authors:** Alex Hinga

**Affiliations:** KEMRI Wellcome Trust Research Programme, Kenya

Thank you for these comments and suggestions.

 Please find our positionality statement, now added to the methods.

Further comments and Responses

*Page 3. During the early stages of the HIV epidemic, placebo-controlled trials of antiretroviral treatment presented ethical tensions because the treatment was only available in developed countries.- please clarify whether the ethical tensions arose because of the nature of placebo-controlled trials or because treatment was only available in developed countries?*

**Response:** The ethical tensions were both because of unavailability of treatments and around the trial design, particularly the use of placebos for an emerging infectious disease with high mortality risk. We have edited the paragraph to emphasize this point.

*Page 5.The CSC and NRC reviewers’ comments and interview transcripts were uploaded as MS Word documents into NVivo12 software (QDA Miner Lite is a freely available alternative software). It is not clear why they have talked about QDA Miner Lite being freely available when they used NVivo - please explain.*

**Response:** Since NVivo12 is a proprietary software, the editorial team requested us to recommend a freely available software that readers/reviewers can use to replicate our study, we recommend QDA Miner Lite as one of the alternatives to Nvivo12.

*The summary of research protocols reviewed by KWTRP is confusing & needs to be presented clearly. The abstract talks about 10 new COVID studies & 42 feedback letters. Table 1, indicates 10 new COVID studies and 28 COVID-related amendments. Page 5, talks about 30 COVID related protocols (n=10). Please be consistent. In addition, they have indicated that 66 protocols were reviewed but the sample sizes for COVID studies or amendments don't add up to 66 - I suggest that they either provide COVID only studies or indicate the number of non-COVID studies.*

**Response:** Thank you for asking us to clarify these important issues. Each of the 10 new COVID-19 protocols was reviewed by at least two people at CSC and NRC. This amounted to a total of of 42 review feedback letters across the 10 protocols. We have now edited the text at the beginning of the Results section to clarify what we mean by COVID-19 related protocols and non-COVID-19 protocols. As shown in Figure 2, there were 30 COVID-19 related protocols and 36 non-COVID-19 protocols. After Figure 2, the rest of the article focuses on the 30 COVID-19 related protocols only. Seven out the 10 new protocols were amended once, 17 ongoing protocols had one COVID-19 related amendment each, while two received two amendments.

*Page 5 & 6, rather than talking about the departments that submitted protocols, readers may be more interested in an overview of the COVID-19 studies reviewed to appreciate the ethical issues presented thereafter.*

**Response:** Thank you for this suggestion: We have now included a broad overview of the types of studies reviewed (as seen below). Naming the studies would risk identifying our study participants.

 “Overall, the protocols outlined studies for investigating the biomedical, clinical, epidemiological and ethical aspects of COVID-19. These included protocols for COVID-19 trials, epidemiological modeling, health care management, policy response and cost-effectiveness of COVID-19 testing. The studies involved a wide range of participants, including health care workers, community members, researchers and policymakers, and employed qualitative and quantitative approaches, including collection of blood samples and interviewing participants.”

*Page 6 & 9, the subtitles/themes are connected to the first paragraph/sentence*

**Response:** Corrected

*Figure 5, has symbols in the first 2 text boxes, immediately after Next step-include the meanings of these symbols in the key.*

**Response:** Thank you for noting this. It has been amended.

*Page 9, it showed that PIs took considerable time to respond to NRC’s comments with median of 24 days (range 0–87 days) for new COVID-19 protocols and 18 days (median of 0–51 days) for amendments. Please check and ensure that the median in calculated properly.*

**Response:** Thank you checking on this. We used Median and IQR because they are the preferred measures of central tendency for skewed distribution. We have now included an extended data file showing the actual number of days taken by reviewers and researchers.

1. *Page 12, some words are in italics please check if this was meant to be.*  

**Response:** Thank you, this has been corrected

1. *Please ensure that COVID-19 and KEMRI-Wellcome Trust Research Programme are spelt consistently. *

**Response:** Thank you. We have corrected this, including in Figure 2 *.*

# Peer review: Reviewer response for version 1

**DOI:** 10.21956/wellcomeopenres.19387.r49045

**Published:** 2022-03-24

**Authors:** Sonali Kochhar

**Affiliations:** 1 Department of Global Health, University of Washington, Seattle, WA, USA; 2 Global Healthcare Consulting, New Delhi, Delhi, India

The article is very relevant as this has been a common concern among researchers that Ethics committees (EC) review will delay trial approval, especially if there is a large volume of reviews, as seen during the pandemic. This article will help other ECs in LMIC to improve their systems and review processes.

- Abstract: Results- Joint review process- it is not clear what the joint review refers to in the abstract as it is not mentioned earlier (it is described in detail in the article).
- In figure 4, please mention the full form of the abbreviation CSC.
- In figure 4, for COVID related protocols, the thought was that 1 week would be saved? (4 weeks for non COVID protocol review vs 5 days + 2 weeks)?  
- Instead of 3 weeks as planned, it took 4 months? This could be clearly clarified.
- This joint review was only done for 1 trial? Please clearly mention what was done for the other trials.
- "Used these terms interchangeably"- please specify what terms.
- "Pre-approved protocols"- what does this mean? How can the protocols be pre-prepared?

Is the work clearly and accurately presented and does it cite the current literature?

Partly

If applicable, is the statistical analysis and its interpretation appropriate?

Yes

Are all the source data underlying the results available to ensure full reproducibility?

Yes

Is the study design appropriate and is the work technically sound?

Yes

Are the conclusions drawn adequately supported by the results?

Yes

Are sufficient details of methods and analysis provided to allow replication by others?

Yes

Reviewer Expertise:

Vaccines (including COVID-19) clinical research, ethics and safety  research, Pandemic and Epidemics preparedness, Health systems strengthening

I confirm that I have read this submission and believe that I have an appropriate level of expertise to confirm that it is of an acceptable scientific standard.

# Response

**Published:** 2022-06-14

**Authors:** Alex Hinga

**Affiliations:** KEMRI Wellcome Trust Research Programme, Kenya

Thank you for these comments and suggestions.

*Abstract: Results- Joint review process- it is not clear what the joint review refers to in the abstract as it is not mentioned earlier (it is described in detail in the article).*

**Response:** Thank you for pointing out this potential lack of clarity. We have now edited the abstract to include this phrase:

  “…and trialing a joint review process where one protocol was submitted to multiple review committees simultaneously.”

*In figure 4, please mention the full form of the abbreviation CSC.*

**Response:** We have now written out “CSC” in full.

*In figure 4, for COVID related protocols, the thought was that 1 week would be saved? (4 weeks for non-COVID protocol review vs 5 days + 2 weeks)?*

**Response:** Before COVID-19, the expectation was that protocol review by institutional and national review committees would take at least 10 weeks (2 weeks at CSC and 2 months at National Review Committee), as outlined in figure 1. Therefore, the expedited review process for COVID-19 protocol during the pandemic sought to save about 7 weeks. Review of non-COVID protocols was paused during the pandemic and when this resumed, it followed the process outlined in figure 1. In figure 4, we have only compared protocol review within KWTRP  before and during the pandemic.

*Instead of 3 weeks as planned, it took 4 months? This could be clearly clarified.*

**

**Response:** Figure 5 illustrates one protocol that took four months from submission to approval. This was included to map and better understand the journey of a protocol from submission to completed review. We have edited the text to clarify that figure 5 refers to the review process for only one protocol. Table 1 shows the median turnaround review time for new COVID-19 protocols and COVID-19 related amendments. The 3-weeks target was not met for any of the new COVID-19 protocols.

*This joint review was only done for 1 trial? Please clearly mention what was done for the other trials.*

**Response:** All the other studies were submitted, reviewed and approved sequentially, starting with the CSC, followed by NRC  then other relevant review and regulatory bodies outside KWTRP depending on type of study.

*"Used these terms interchangeably"- please specify what terms.*

**Response:** The terms are ‘quick turn-around’ and ‘expedited review’

*"Pre-approved protocols"- what does this mean? How can the protocols be pre-prepared?*

**Response:** Thank you for raising this point. The WHO guidance for managing ethical issues in infectious disease outbreaks suggests that to enhance review in time-sensitive circumstances, there could be “advance review of generic protocols for conducting research in outbreak conditions, which can then be rapidly adapted and reviewed for particular contexts.” Some participants supported this recommendation and explained why this would have strengthened research review during COVID-19. We have added an illustrative quote [Last Paragraph of Results Section that might clarify this concept.
